# Diagnostic Value of Circulating microRNAs for In-Stent Restenosis in Patients with Lower Extremity Arterial Occlusive Disease

**DOI:** 10.1038/s41598-018-36295-2

**Published:** 2019-02-04

**Authors:** Liangxi Yuan, Jian Dong, Guanglang Zhu, Junmin Bao, Qingsheng Lu, Jian Zhou, Zaiping Jing

**Affiliations:** Department of Vascular Surgery, Changhai Hospital, Second Military Medical University, Shanghai, 200433 P.R. China

## Abstract

In-stent restenosis (ISR) is still a major cause of failure of endovascular stenting treatment in patients with lower extremity arterial occlusive disease (LEAOD). Sensitive and reliable biomarkers for early diagnosis to predict ISR should be considered. This study was conducted to explore the diagnostic value of microRNA in predicting ISR in patients with LEAOD after endovascular stenting treatment. From March 2014 to July 2016, 208 patients (170 males and 38 females) with LEAOD undergoing interventional treatment were enrolled in this research. Patients were divided into the restenosis and non-restenosis groups according to routine postoperative angiography. Circulating microRNAs expression were detected in 208 participants, including 78 ISR patients, 68 non-ISR patients and 62 healthy volunteers. We selected 6 microRNAs from microarray screening as candidates for further testing via qRT-PCR. A receiver operating characteristic (ROC) curve was generated to assess the diagnostic value of circulating microRNAs in predicting ISR for LEAOD patients. The results showed that circulating microRNA-320a and microRNA-572 in patients with ISR (n = 78) had significantly higher expression levels than it from non-ISR and healthy volunteers. By receiver operating characteristic curve analysis, the sensitivity was 82.1% and the specificity was 63.8% for microRNA-320a; the sensitivity was 69.2% and the specificity was 68.9% for microRNA-572, and the AUC was 0.766 and 0.690 for detection of ISR, respectively. Furthermore, 78 patients with ISR had significantly higher circulating expression levels of microRNA-3937 and microRNA-642a-3p and lower circulating expression levels of microRNA-4669 and microRNA-3138 compared with 68 non-ISR patients and 62 healthy volunteers, but they have no significant difference. We found that differential circulating microRNA expression in patients after stenting with ISR, and the data indicate that circulating microRNA-320a and microRNA-572 have promising value in diagnosing ISR in patients with LEAOD.

## Introduction

Currently, endovascular stenting treatment, such as self-expanding nitinol stents treatment, has become one of the primary treatment in patients with obstructive atherosclerotic disease and have been shown to be superior to percutaneous transluminal angioplasty (PTA)^1^. The use of drug-eluting stents (DESs) has drastically reduced the incidence of in-stent restenosis (ISR), as compared with standard balloon angioplasty (POBA) in patients with superficial femoral artery diseases^2^. In spite of these advantages, ISR is still an important clinical problem. Therefore, a sensitive and reliable biomarker for early diagnosis to predict ISR in patients with lower extremity arterial occlusive disease after stenting would be ideal.

MicroRNAs (microRNAs) are a kind of small (approximately 22 nucleotides), single-stranded noncoding RNAs, which control various biological functions and play important roles in physiologic and pathologic processes by inducing mRNA degradation and translation interruption^3^. Increasing evidence indicates that microRNAs regulate diverse cellular functions, such as proliferation^4^ and apoptosis^5^. Therefore, these microRNAs must have been association with the development of cardiovascular diseases. Zahedi *et al*. proposed that Dicer expression plays a vital role by reducing smooth muscle cells (SMCs) proliferation in vascular repair after vascular injury by generating anti-proliferative microRNAs, such as microRNA-27a-3p, to prevent vessel stenosis due to exaggerated neointima formation^6^. Some studies have shown that microRNAs can stably exist in the circulating blood and can be used as a biological marker for the diagnosis of disease^7^. Circulating miRNA-21, miRNA-143, miRNA-145, and miRNA-100 showed differences between ISR patients groups and non-ISR patients groups, and these miRNAs were closely related to the occurrence of ISR after drug eluting stent implantation in coronary artery disease (CAD) patients^8,9^. Yu *et al*. detected serum microRNA-143 expression by using quantitative real-time polymerase chain reaction (qRT-PCR) and found that microRNA-143 can be a promising tool for predicting the ISR in patients with lower extremity arterial occlusive disease^10^. The purpose of this study was to investigate the expression of circulating microRNA from microarray screening and qRT-PCR and assessed its diagnostic value in patients after stenting with and without ISR, and to provide evidence for early detection of restenosis after stenting.

## Materials and Methods

### Ethics statement

This study was approved by the Ethical Committee of the Affiliated Changhai Hospital of Second Military Medical University, and we confirmed that all research was performed in accordance with relevant guidelines and regulations. All the participants have signed the informed consent before enrollment.

### Population

From March 2014 to July 2016, 208 consecutive symptomatic patients (170 males and 38 females; mean age, 77 years; range, 68–84 years) with LEAOD who underwent treatment in the Department of Vascular Surgery of Shanghai Changhai Hospital of Second Military Medical University were enrolled in this study. All patients were diagnosed with LEAOD at different levels based on computed tomography angiography (CTA). The inclusion criteria were as follows: (1) major lesions of femopopliteal arteries; (2) at least one distal run-off below the knees after the intervention. Exclusion criteria were as follows: (1) patients who refused to undergo Doppler ultrasound review; (2) patients who did not take anti-platelet aggregation drugs after surgery; (3) patients who had ISR in two or more locations and received revascularization therapy within 1 month after the diagnosis of ISR; (4) patients who had connective tissue diseases, or malignancy.

### Sample collection and storage

Blood samples for microRNAs detection were obtained from all patients and were processed within 1 hour of collection by two-step centrifugation. Plasma was transferred to 1-mL nuclease Eppendorf (EP) tubes and refrigerated in an ultra-low-temperature refrigerator at −80 °C.

### RNA Isolation

Total RNA was taken out from plasma using the microRNA Neasy Mini Kit (Qiagen, Hilden, Germany) in accordance with the instructions from the manufacturer. The concentration and purity of the RNA solution were detected by NanoDrop ND-2000 Spectrophotometer (Thermo Scientific Wilmington, DE, USA). The samples absorbance at 260/280 and at 260/230 nm were used for further analyses. All of the purified RNA samples were stored at −80 °C for further processing.

### Microarray

The expression of candidate microRNAs was measured in the plasma of every sample by human MicroRNA Microarrays (Release 16.0, 8 × 60k) (Agilent Technologies, Santa Clara, CA). The microRNAs samples were labeled by the Complete Labeling and Hyb Kit (Agilent Technologies, Santa Clara, CA, US), and hybridized with the Cy3-tagged RNA. Quantile algorithm (Gene Spring Software 12.6) was used to normalize the raw data. The prediction analysis of microarrays (PAM) was used for microarray analysis.

### Quantitative real-time PCR

Quantitative reverse transcriptase-polymerase chain reaction (qRT-PCR) was carried out to test the candidate microRNAs acquired on microarray. The RNA sample with high quality was used for reverse transcribed reaction, which was performed by the TaqMan microRNA Reverse Transcription Kit (Applied BioSystems, Foster City, CA). Then qRT-PCR reaction was carried out by Taqman microRNA assays (Applied Biosystems, Foster City, CA) in an ABI PRISM 7900HT Sequence Detection System (Applied Biosystems, Foster City, Calif., U.S.A.). The total volume of the reaction was 10 μL, including 2 μL cDNA template, 5 μL of Universal Master Mix (Applied Biosystems, Foster City, Calif., U.S.A.) and 3 μL water. The primers used in our study were designed in accordance with the sequences of the candidate microRNAs. All reaction mixtures were incubated in a 96-well optical plate at 95 °C template denaturation for 10 min, followed by 40 cycles at 95 °C for 15 seconds and at 60 °C for 1 minute. Each reaction was repeated in triplicate. The microRNAs which showed Cycle threshold (CT) values correspond to the number of cycles required for the amplified product to reach a critical threshold of detection. The circulating levels of microRNAs were analyzed by two investigators who were blinded to the clinical data of patients.

### Follow up

Technical success was defined as reestablishment of direct flow to the distal artery with residual stenosis of <30% in the femoral arteries. Clinical success was defined as a subjective perception of improved walking distance, resolution of resting pain, or healing of ischemic ulcer, resulting in at least one category improvement based on the Rutherford classification together with an increase in ankle brachial index (ABI). Patency was determined by duplex ultrasonography in all patients, and contrast-enhanced computed tomography was performed if clinically indicated. Restenosis was defined as >2.4 of peak systolic velocity ratio determined by duplex ultrasonography.

### Statistical Analysis

SPSS 18.0 statistical software was used for data analysis. The data continuous variables are summarized and presented as mean ± standard deviation (SD) or median. One-way ANOVA was used to assess the differences among the three cohorts. The categorical variables among the three groups were assessed using Chi-square tests. Receiver operating characteristic (ROC) analysis by statistical software was used to evaluate the diagnostic performance of the potential microRNAs. P < 0.05 was considered statistically significant.

## Results

### Characteristics of Study Population

A total 208 participants, including 78 ISR patients, 68 non-ISR patients and 62 healthy volunteers were enrolled in this study and followed up. The baseline characteristics of the study subjects are compared in Table 1. There were no significant differences in age, gender, hypertension, or hyperlipidemia between the restenosis group and the control groups (all P > 0.05).

**Table 1:** Patient Characteristics.

Characteristics	ISR (n = 78)	Control(n = 130)		χ^2^/t	P
		non-ISR (n = 68)	Healthy (n = 62)		
Gender male	67(85.9%)	53(77.9%)	50(80.6%)	1.451	0.228
Age, years	66.32 ± 7.70	65.46 ± 13.80	67.01 ± 4.80	0.035	0.851
Smoking	30(38.5%)	35(51.5%)	20(32.3%)	0.298	0.585
Diabetes	56(71.8%)	47(69.1%)	31(50%)	2.959	0.085
Hypertension	46(59.0%)	40(58.8%)	37(59.7%)	0.001	0.971
Atrial fibrillation	4(5.1%)	3(4.4%)	3(4.8%)	0.028	0.867
CAD	18(23.1%)	16(23.5%)	12(19.4%)	0.067	0.796
Cerebral infarction	10(12.8%)	3(4.4%)	8(12.9%)	1.020	0.312
COPD	5(6.4%)	3(4.4%)	2(3.2%)	0.700	0.403
Cholesterol, mmol/L	4.31 ± 1.05	4.47 ± 1.12	4.57 ± 1.54	0.972	0.325
LDL, mmol/L	2.38 ± 0.85	2.49 ± 0.99	2.21 ± 0.85	0.713	0.399
HDL, mmol/L	1.03 ± 0.40	1.10 ± 0.26	1.12 ± 0.38	2.896	0.090

Abbreviations: CAD, Coronary artery disease; COPD, Chronic Obstructive Pulmonary Disease; LDL, Low Density Lipoprotein; HDL, High Density Lipoprotein.

Continuous data are presented as the means ± standard deviation;

Categorical data are given as the counts (percentage).

### MicroRNAs Screening

**Figure 1:**
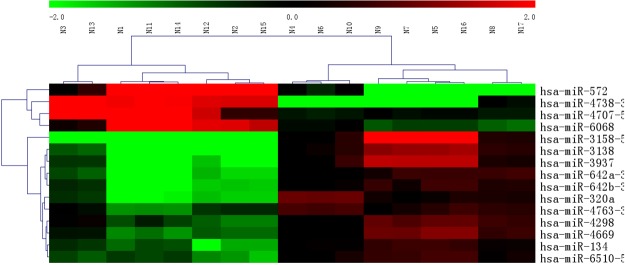
Microarray results of different microRNAs in the peripheral blood of enrolled patients.

**Figure 2:**
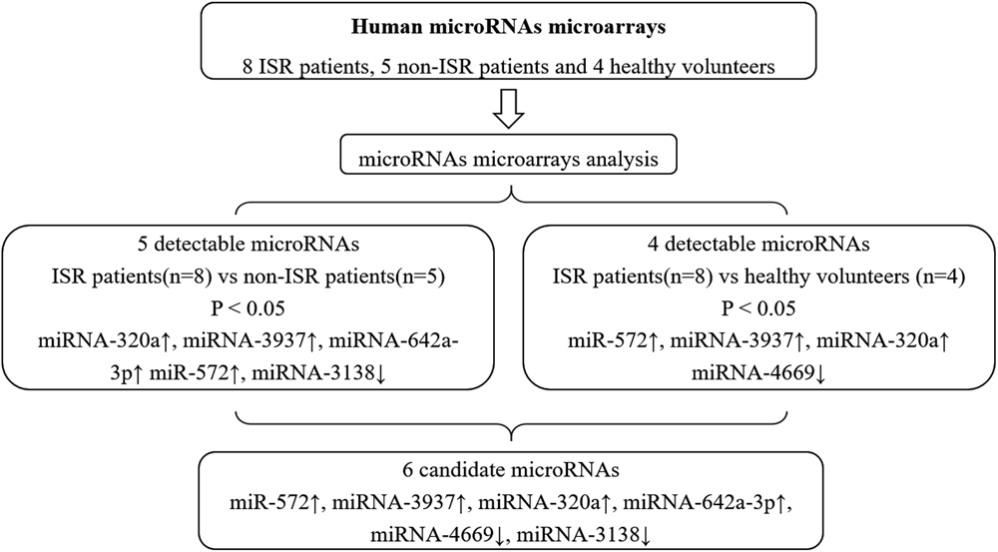
Process for Selection of Candidate MicroRNAs, and 6 candidate microRNAs discovered via microarrays were selected for the further validation.

To observe the differential expression levels of microRNAs among patients with ISR, non- ISR and healthy volunteers, we performed gene expression microarray analysis. The microarray analysis showed significant changes in microRNAs, which were up-regulated or down-regulated in ISR groups compared with non- ISR and healthy volunteers (Fig. 1). All assays of samples from 8 ISR patients, 5 non-ISR patients and 4 healthy volunteers were successfully conducted on plasma specimens used to screen the significant differential expression levels of microRNAs with the microarray containing probes. As shown in Fig. 2, expressions of plasma microRNA-320a, microRNA-3937, microRNA-642a-3p and microRNA -572 were significantly higher in ISR patients than in control groups. In contrast, microRNA-4669 and microRNA-3138, exhibited significantly lower expression levels in the ISR group than that in the control groups. Then the 6 differentially expressed microRNAs were identified as candidates and were tested using the entire sample set (78 ISR patients, 68 non-ISR patients and 62 healthy volunteers) with qRT-PCR, and all the 6 microRNAs passed quality control. Among the microRNAs, microRNA-320a and microRNA-572 had a significantly different pattern between ISR groups and control groups (P < 0.001).

### MicroRNAs Training with qRT-PCR

The 6 candidate microRNAs were tested using the entire sample set with qRT-PCR, and all of them passed quality control. Among the microRNAs, microRNA-320a and microRNA-572 had a significantly difference between the ISR groups and control groups (P < 0.001). While there was no significant difference in microRNA-3937, microRNA-642a-3p, microRNA-4669 and microRNA-3138 between the ISR groups and control groups. The results are summarized in Table 2.

**Table 2:** Characteristics of the Subjects by Real-Time PCR.

Characteristics	ISR (2^−△Ct^) (n = 78)	Control (2^−△Ct^) (n = 130)		χ^2^/t	P
		non-ISR (n = 68)	Healthy (n = 62)		
miR-642a-3p	4.45 ± 2.39	3.77 ± 2.19	3.94 ± 2.51	1.666	0.192
miR-3937	3.79 ± 1.99	3.56 ± 2.05	3.50 ± 2.27	0.379	0.685
miR-4669	3.86 ± 2.33	4.15 ± 2.58	4.45 ± 2.23	1.081	0.341
miR-3138	3.60 ± 2.25	3.63 ± 2.46	3.97 ± 2.76	0.459	0.633
miR-320a	4.80 ± 1.90	3.26 ± 1.58	3.06 ± 1.70	21.858	<0.001
miR-572	4.85 ± 1.90	3.30 ± 1.80	3.92 ± 2.05	12.152	<0.001

### MicroRNA Levels in ISR Patients

**Figure 3:**
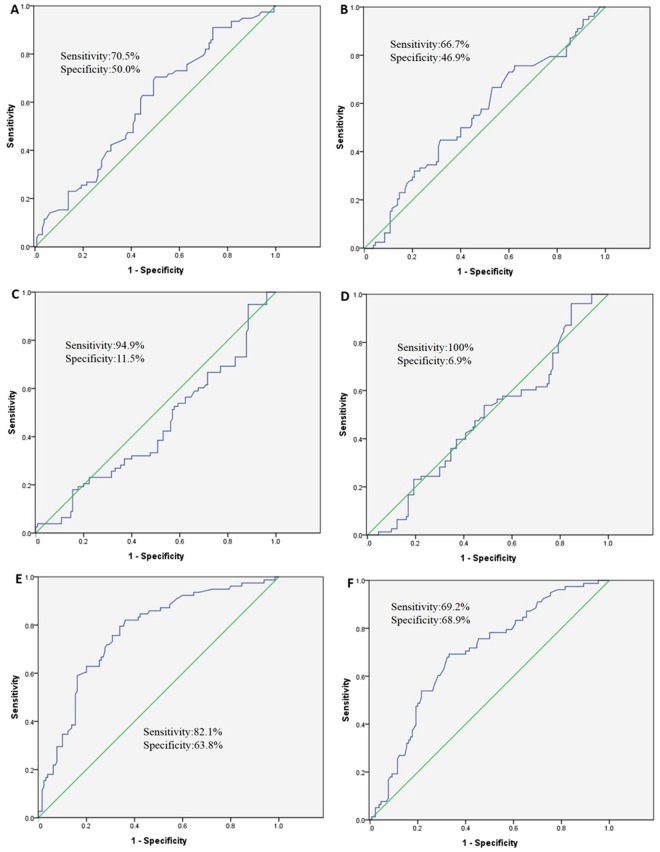
Receiver operating characteristic (ROC) curve analysis with respect to microRNA-642a-3p (**A**), microRNA-3937 (**B**), microRNA-4669 (**C**), microRNA-3138 (**D**), microRNA-320a (**E**), microRNA-572 (**F**).

ROC analysis was conducted in the 6 candidate microRNAs between ISR and control groups (Table 3). ROC analysis for those four microRNAs (microRNA-3937, microRNA-642a-3p, microRNA-4669 and microRNA-313) exhibited sensitivity of 70.5%, 66.7%, 94.9% and 100%, respectively. While specificity of them were 50.0%, 46.9%, 11.5% and 6.9%, respectively. The corresponding AUCs were 0.591, 0.554, 0.439 and 0.487(Fig. 3A–D). The ROC analysis showed an optimal cutoff value for microRNA-320a with a sensitivity of 82.1% and a specificity of 63.8%, and the corresponding AUC was 0.766 (Fig. 3E). The sensitivity, specificity, and AUC of microRNA-572 were 69.2%, 68.9%, and 0.690, respectively (Fig. 3F). The ROC analysis suggested the microRNA-320a and microRNA-572 could distinguish ISR from non- ISR subjects and healthy subjects. In addition, microRNA-320a and microRNA-572 could be a promising tool for predicting ISR in LEAOD patients.

**Table 3:** ROC Analysis for MicroRNA.

microRNA	ROC analysis		
	Sensitivity	Specificity	AUC
miR-642a-3p	0.705	0.500	0.591
miR-3937	0.667	0.469	0.554
miR-4669	0.949	0.115	0.439
miR-3138	1.000	0.069	0.487
miR-320a	0.821	0.638	0.766
miR-572	0.692	0.689	0.690

## Discussion

The present study is to investigate the prognostic value of microRNA in predicting ISR in patients with LEAOD undergoing peripheral angioplasty with endovascular stenting treatment. Endovascular treatment by stent implantation may be considered a first-line treatment option for LEAOD with significant effective, safety and less trauma^11^. However, the incidence of ISR restricts the development of the endovascular techniques and ISR is the most frequent and major problem after stent implantation for treatment of LEAOD^12^. Although some researchers have attempted to prevent the occurrence of ISR, we still do not have an appropriate treatment for ISR^13^. It is an urgent problem to solve ISR in the treatment of LEAOD and increasing attention has been directed toward the correlative factors of ISR in LEAOD after stent implantation.

The key pathophysiologic phenomenon of restenosis after stenting is due to the generation of neointima, which is essentially caused by the proliferation of VSMCs and subsequent abluminal migration^14^. Gareri C *et al*. focused on microRNAs, which are involved in the regulation of SMCs migration and proliferation^15^. In addition, they explored the microRNAs related to the platelet activation and endothelial dysfunction, which can lead to both stent thrombosis and in-stent restenosis. Endothelial-derived microRNAs are involved in the modulation of neointima formation enhancement and endothelial recovery impairment. To date, several miRNAs have been shown to participate in the regulation of angiogenesis in the context of endothelial cells. The previous study reported that miR-16 has been shown to be differentially expressed in endometriosis, in which angiogenesis may be involved in the growth of the endothelium and miR-16 participates in the inhibition of angiogenesis in multiple ways^16^. Sorrentino *et al*. found that endothelial miR-16 is remarkably upregulated after vascular injury in hind limb ischemia and shows a negative effect on endothelial repair and remote vascular remodeling at the carotid artery^17^.

Blood-based biomarker test is convenient, low cost, fast and effective that may be a promising approach for routine screening. In this study, we found that the levels of circulating microRNAs were significantly altered in ISR patients. In particular, our microRNA screening revealed that patients with ISR had significantly differential expression levels of microRNA-320a, microRNA-572, microRNA-3937, microRNA-4669 and microRNA-3138 and microRNA-642a-3p when compared with control groups. From entire sample set testing with qRT-PCR and ROC analysis, the patients with ISR had significantly higher expression levels of microRNA-320a and microRNA-572, reveling their highest diagnostic accuracy for detection of ISR in patients with LEAOD.

In earlier studies, microRNAs are known as vital regulators for many cellular mechanism and some researches have showed that circulating microRNAs are related to ISR in peripheral and coronary artery disease. Plasma microRNA-143 and microRNA-145 were reported to be the potential biomarkers for in-stent restenosis^8^, which promoted SMCs proliferation, migration, and neointima formation^18,19^. Also, microRNA-143 was reported to play a vital role in modulating VSMC phenotypes^20^, and microRNA-143 has proposed to be used for their diagnosis and prognosis for postoperative restenosis^10^. Circulating microRNA-195 predicts target lesion restenosis, atherothrombotic events and target vessel revascularization after infrainguinal angioplasty with stent implantation in patients with peripheral artery disease^21^. MicroRNA-133a is associated with a higher incidence of major adverse cardiac events in patients with CAD. The differential modulation of miR-133a release in the coronary circulation may reflect pathophysiological mechanism involved in CAD complications and suggest a novel potential role in the development of in-stent restenosis^22^. However, in the present study, microRNA-145, microRNA-195, microRNA-133a and microRNA-143 were undetectable in almost half of the patients with LEAOD, which were significantly different from cardiovascular diseases. Therefore, these microRNAs may not be suitable as biomarkers for risk stratification of PAD patients, although they have an important role in pathophysiology of restenosis and could represent interesting therapeutic targets^15^.

Numerous evidences indicated that microRNAs showed evidence of vital role in regulating gene expression, which are involved in the pathological process of cardiovascular diseases^23–25^. More specifically, microRNA -320a plays functional roles in regulating the vascular endothelial cell function via targeting neuropilin 1^26^. It has been reported that microRNA -320a plays an important role in the progression of atherosclerosis and endothelial apoptosis^27^. It has been proved that the over-expression of microRNA-320a in ischemic cardiomyopathy and aortic stenosis^28^. Subsequently, microRNA-320a expression is reported to be associated with potential cardiovascular target genes^29^. The human microRNA-572, in particular, has a length of 20 nucleotides and is located in chromosome 4^30^. Recent studies have found that the level of microRNA-572 in blood is altered in different human diseases, such as postoperative cognitive dysfunction^31^, multiple sclerosis^32,33^, ovarian cancer^34^ and nasopharyngeal carcinoma^35^. However, we did not find any different expression of microRNA-572 in cardiovascular diseases from former literatures and it was the first time to find the different levels of microRNA-572 in the patients with LEAOD.

Our study has some limitations. First, the sample size of enrolled patients was not very large and a larger sample with a longer follow-up is needed to confirm the diagnostic role of these microRNAs in patients with LEAOD. Second, many factors, such as lifestyle and dietary factors, may affect the incidence of ISR. Third, a future multicenter, randomized, prospective study should be designed and performed to illustrate the relationship between microRNAs and ISR.

## Conclusions

We found that differential circulating microRNA expression in patients after stenting with ISR, and the data indicate that circulating microRNA-320a and microRNA-572 have promising value in diagnosing ISR in patients with LEAOD. Larger sample with longer follow-up is needed to confirm the diagnostic role of these microRNAs in patients with LEAOD.
